# Evaluating Malnutrition Practices and Mother’s Education on Children Failure to Thrive Symptoms Using Entropy-Weight and TOPSIS Method

**DOI:** 10.3390/children11080903

**Published:** 2024-07-26

**Authors:** Maria Tzitiridou-Chatzopoulou, Georgia Zournatzidou, Eirini Orovou, Maria Lithoxopoulou, Eftychia Drogouti, George Sklavos, Evangelia Antoniou, Christos Tsakalidis

**Affiliations:** 1 School of Healthcare Sciences, Midwifery Department, University of Western Macedonia, 50100 Kozani, Greece; eorovou@uniwa.gr; 2 Department of Business Administration, University of Western Macedonia, 51100 Grevena, Greece; aff01667@uowm.gr; 3 Neonatal Intensive Care Unit, 2nd Neonatal Department, Aristotle University of Thessaloniki, “Papageorgiou” General Hospital of Thessaloniki, 54635 Thessaloniki, Greece; lithoxopoulou@auth.gr (M.L.); edrogouti@auth.gr (E.D.); golema@auth.gr (C.T.); 4 Department of Business Administration, University of Thessaly, 41500 Larissa, Greece; gesklavos@uth.gr; 5 Department of Midwifery, School of Health & Care Sciences, University of West Attica, 12243 Athens, Greece; lilanton@uniwa.gr

**Keywords:** malnutrition, failure to thrive, Sub-Saharan Africa, children, growth disorder maternal education

## Abstract

Background/Objectives: Failure to thrive (FTT) is mostly caused by insufficient consumption of nutrient-rich food, recurrent infections like diarrhea and intestinal worms, substandard caregiving practices, and limited availability of health and other vital services. Furthermore, there was a correlation between the educational level of mothers and the occurrence of FTT in children aged 6–12 months. Thus, the objective of the current research is twofold: (i) to investigate other factors related to FTT and (ii) to evaluate the impact of them on FTT in Sub-Saharan African countries and their urban areas. Methods: We used weight entropy and TOPSIS methods to approach the research question. In particular, the entropy-weight method is effective for precisely evaluating the relative significance of the selected criteria for TOPSIS computation. Thus, data were retrieved from the database of UNICEF for the year 2019 for nine Sub-Saharan countries, and based on the methods used, five criteria have been selected for consideration. Those of mothers in higher education were identified as having a higher weight, which means that this can affect positively the ability of mothers to mitigate the situation of FTT and protect their children. Results: The findings of the study highlight the factors of maternal education at a higher level and unhealthy habits as those with the greatest weight and impact on the FTT. Moreover, the results indicate that the association between maternal education, and especially higher education, and FTT is stronger in Ethiopia. Despite the limited amount of research on the specified relationship in Sub-Saharan countries, this study is among the initial ones to examine it. Conclusions: The current study can aid policymakers in devising appropriate policies and implementing effective measures to tackle FTT in Sub-Saharan Africa, like enhancing the number of mothers in these countries to be integrated into the educational system to help both themselves and their children mitigate or avoid the symptoms of FTT.

## 1. Introduction

The primary concern of a mother is to provide nourishment and facilitate the growth of her baby. Ensuring the well-being and monitoring the development of the infant is an essential duty that every parent aspires to achieve. Most mothers want nothing less than the utmost quality for their children. Children possess an intrinsic propensity to expand, develop, and achieve progress [1]. They have an inherent tendency to thrive and succeed. Unfortunately, a substantial proportion of infants globally are denied this crucial right. When difficulties occur and impede the growth of the baby, it is a substantial and profound difficulty for the parents. It is crucial to consider the importance of classifying this circumstance as failure to thrive (FFT) [2].

Specifically, FTT or weight faltering is a term that describes the insufficient weight development of juvenile patients. For children, FTT can be characterized by weight loss, which may be defined as (i) weight for age that is below the fifth percentile on standardized growth charts, (ii) a weight percentile decline of more than two major percentile lines on the growth chart, or (iii) a weight for height–length ratio that is below the 80th percentile of the median. Studies have shown that children under the age of five in underdeveloped nations are hindered from achieving their maximum capabilities due to exposure to various risk factors. Poverty significantly influences mental health, especially in early children, but it also has a substantial impact on individuals in general [3]. Early-life malnutrition has significant implications for brain development, as shown by the research conducted by Grantham-McGregor et al. in 2007 [4,5,6,7,8,9,10]. This may have a detrimental impact on children’s future capacity to acquire knowledge and succeed academically. The cognitive capacity of individuals who fail to flourish over a lengthy period should not be underestimated, as research suggests it may result in a decrease of around four IQ points.

Infant and child health providers should understand that malnutrition is not just caused by insufficient food intake or physical sickness. Mothers’ education may also contribute. Studies show that moms’ education levels affect their children’s health [11,12,13,14]. Children of educated moms are less likely to be underweight, wasting, or stunted [4,15,16]. Studies in Jamaica, Bolivia, and Kenya have linked maternal education to infant nutrition [17]. However, the mechanisms that link a mother’s education to her child’s health remain unknown. Glewwe (1999) suggests three ways schooling may affect children’s health [4]. Early formal education of mothers gives health knowledge to succeeding mothers [17,18].

Additionally, women’s education increases their reading and math skills, and their ability to spot ailments and seek medical treatment for their children. Additionally, they are better at understanding and following pediatric medical recommendations [19]. Longer education increases women’s receptivity to modern medicine. Research has established a strong link between mothers’ education, socioeconomic status, and children’s nutrition. Higher-educated women tend to find steadier and more rewarding jobs. They also marry more educated and wealthy people. These educated mothers also live in wealthier communities, which affects their children’s health and survival. Education also affects moms’ depression risk, according to research. Also, maternal depression has been connected to child health issues, including malnutrition [20,21].

Against this background, this study tries to evaluate the impact of unhealthy feeding and mothers’ education on FFT infants aged 6–12 months who live in urban areas of ten African countries, which are indicated as those with the highest level of FFT symptoms. To approach the main objective of the study, the entropy-weight and TOPSIS methods have been applied. The novelty of this study is the consideration of both malnutrition practices that refer to the zero intake of fruits and vegetables by children and to the mothers’ education level (primary, secondary, higher, and non-education). The findings of the current study can help health practitioners in the field mitigate FFT symptoms in children in Africa, as the prevalence of FFT among children in the same age category is 30 percent, which is significantly higher than the global estimate of 22.3 percent.

This research is built as follows. Section 2 reviews the literature on African FFT symptoms and enhancement factors. Section 3 shows materials and techniques, while Section 4 presents research results. Research findings, consequences, and limitations are discussed in Section 5.

## 2. Failure to Thrive in Sub-Saharan Africa: The Role of Mothers’ Education Knowledge toward Its Mitigation

Childhood malnutrition, which includes stunted development and a lack of essential nutrients, is linked to higher mortality rates and is believed to be responsible for around 45% of fatalities in children under the age of five worldwide [15,22]. Although there have been decreases in the rates of stunting, wasting, and underweight in almost all African nations from 2000 to 2015, several regions in Sub-Saharan Africa still have a high incidence of stunting, with rates as high as 30% [23,24]. The WHO Global Nutrition Targets for the period from 2012 to 2025 have the objective of decreasing the occurrence of stunting by 40% and reducing the occurrence of wasting to below 5%. Several nations in Sub-Saharan Africa are projected to meet these objectives by 2025, but only a small number are anticipated to accomplish Sustainable Development Goal 2.2, which is to eradicate malnutrition by 2030.

**Figure 1:**
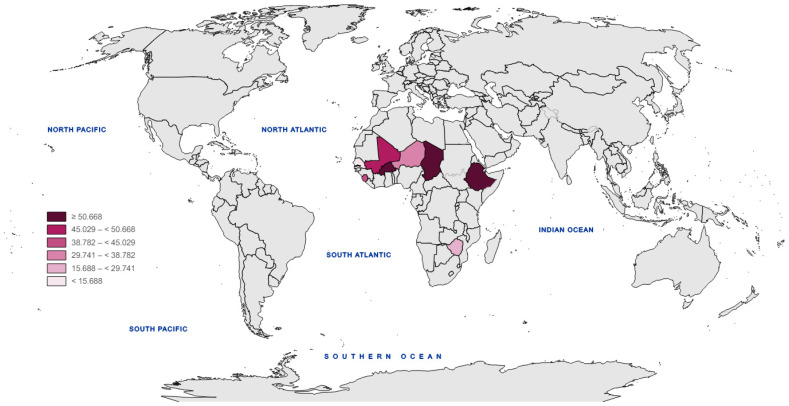
Failure to thrive index in selected Sub-Saharan African countries.

FTT hinders the ability of countries in Sub-Saharan Africa to accomplish the SDGs (Figure 1). This is mostly contingent upon the condition of FTT [25,26]. Also, FTT may be most accurately described as insufficient physical development that is detected by monitoring growth over a period using a standardized growth chart. The National Center for Health Statistics (NCHS) has recently published enhanced growth charts, which may be seen at www.cdc.gov (accessed on 5 May 2024). FTT is often diagnosed by practitioners when a child’s weight for age is below the fifth percentile of the standard NCHS growth chart or if it crosses two main percentile lines. A recent study has shown that the weight-for-age strategy is the most straightforward and logical indicator for FTT [27,28]. Additional growth factors that may aid in diagnosing FTT include weight-for-height and height-for-age [29].

FTT is often ascribed to prenatal, genetic, and epigenetic influences, as well as postnatal environmental exposures, such as insufficient diet, contaminated water, and inadequate sanitation and hygiene. Other contributing factors include low parental education, recurrent acute illnesses, and chronic subclinical inflammation of the small intestine. The impact of contaminated water, inadequate sanitation and hygiene, and nutritional treatments on growth outcomes has been varied. Despite efforts to reduce child mortality and diarrheal illness, there has not been a significant drop in stunting, as predicted. Empirical data collected from rural areas of Gambia over the course of the last three years indicate that significant and consistent enhancements in living standards, illness mitigation, dietary practices, and healthcare provisions may be necessary to eradicate undernutrition. However, the causative pathways of FTT in Sub-Saharan are still under investigation. Thus, the current research aims to indicate those factors [1].

Therefore, based on the above diet, nutrition habits and low education knowledge of parents can be categorized as causative pathways of FTT [30]. On the other hand, enhancing the knowledge level of parents, particularly mothers, about nutrition, cleanliness, and techniques for preventing prevalent diseases should logically decrease the rates of malnutrition-related deaths and illnesses. According to popular belief, the key to nourishing a kid is by influencing the mother’s thoughts and beliefs [31,32]. The mother or caregiver has complete control over the quality, variety, and amount of food consumed. This issue is of the utmost importance in Sub-Saharan Africa, where the ability of girls in particular areas to get formal education remains a significant and pressing obstacle. There is a clear correlation between malnutrition and poverty, as well as the quality of food consumed, prevalence of diseases, and overall health state. The correlation between education and poverty is very significant, as it becomes intricately intertwined inside the perpetual cycle of ignorance, sickness, and poverty [29]. Education has the potential to mitigate the prevalence of disproportionately high family sizes, as seen in many areas of Sub-Saharan Africa. A socioeconomically disadvantaged society with certain cultural beliefs may not be aware that having fewer children might help them better manage their limited resources and provide sufficient and high-quality nourishment for their family [27,33,34].

Musgrove et al. elucidate three essential mechanisms via which ignorance and inadequate education exacerbate the problem of starvation. Initially, individuals may possess little knowledge about vitamins or nutrients, resulting in their failure to consume even the inexpensive and easily accessible ones [35]. Furthermore, there is a lack of knowledge on the origins of diseases and their subsequent effects. Treatment and preventative methods are often readily available and affordable. Inadequate sanitation and the inability to manage some intestinal parasites have significant consequences in terms of competing for nutrition with the host, resulting in anemia and lowering appetite. Significant declines in academic achievement have been shown in children who are afflicted with these parasites. Furthermore, there is a possibility that some individuals lack knowledge about the proper care of their young children, leading them to underestimate the importance of healthy behaviors, such as breastfeeding and providing their children with vitamins and other foods that are rich in essential nutrients [36]. The primary factor responsible for the reduction in child malnutrition between 1970 and 1995 was the significant advancements in women’s education, accounting for 43% of the overall decrease. In comparison, increases in per capita food supply contributed around 26% to the decline.

Finally, FTT is not only limited to rural areas. In many African nations with a high prevalence of undernutrition, FTT in urban children under the age of five is a notable concern, but the severity of the condition is not as pronounced as in rural regions. This is not only a matter of wealth disparity [25,26,28]. Evidence suggests that, although undernutrition rates are more prevalent in poorer families in Sub-Saharan Africa, they nonetheless remain quite high among homes that are not classified as poor. In several nations within the area, the prevalence of underweight children exceeds 15% among those belonging to the highest income quintile. Therefore, the current study focuses on investigating the phenomenon of FTT in urban Sub-Saharan African areas to contribute further to the enhancement of the literature and to help policymakers in their decision-making regarding the development of strategies and practices against FTT [29]. Therefore, the current study considers urban areas from nine Sub-Saharan African countries to evaluate the factors that can affect FTT symptoms.

## 3. Materials and Methods

TOPSIS is a systematic approach that takes into account multiple variables within a well-defined framework to facilitate decision-making. Entropy weighting is the procedure of calculating the entropy of different variables or components to assign weights depending on their level of uncertainty or unpredictability [37]. The TOPSIS model is a combination of the entropy and TOPSIS methodologies. The primary goal of the system is to ascertain the importance of each assessment signal by employing the entropy-weight technique. The most effective method for classifying assessment items is the act of locating the item. The entropy-weight TOPSIS technique is based on the fundamental concept of identifying the most optimal feasible solution, where each attribute value has reached its highest or lowest value in the alternative scheme [38,39,40,41]. To determine the optimality of a test item, we compute its distance from both the most favorable and least favorable conditions. An assessment object is deemed optimum when it closely approximates the desired response while being within permissible limits [42,43]. In contrast, it is considered suboptimal if it fails to satisfy these criteria. Conversely, it is deemed suboptimal if it fails to meet these criteria. Furthermore, the TOPSIS approach does not enforce any limitations on the sample size, enabling it to efficiently use the data from the original source. It has the advantages of low loss of information and versatile operation [37,44].

Prior to introducing the method used, we establish the initial presumption that there are n alternatives $A=\left\{A_{1}{,A}_{2}{,A}_{3},\ldots ,A_{n}\}\right.$ and *m* criteria $M=\left\{M_{1}{,M}_{2}{,M}_{3},\ldots ,M_{m}\}\right.$, where $i\in A,j\in M,i=\left\{1,2,3,\ldots ,n\right\},j=\left\{1,2,3,\ldots ,m\right\}$.

The matrix $X^{\prime }={(x^{\prime \prime }}_{ij})$ n × m in Equation (1) is a decision matrix of n×m. The weights of criteria *M* can be represented by the weight vector $W=\left\{w_{1},w_{2},w_{3},\ldots ,w_{m}\right\}$, which satisfy $\sum_{j=1}^{m}w_{j}=1.$

(1) $$X^{\prime }=\left[\begin{array}{ccc} {x^{\prime }}_{11} & {x^{\prime }}_{12} & {x^{\prime }}_{1m}\\ {x^{\prime }}_{21} & \ldots & {x^{\prime }}_{2m}\\ {x^{\prime }}_{n1} & {x^{\prime }}_{n2} & {x^{\prime }}_{nm} \end{array}\right]$$

### 3.1. Entropy-Weight Method

The weight may be determined by the Shannon entropy-weight approach, which considers the degree of data dispersion [45]. Initially, we employ the min–max method to standardize the *n* × *m* original decision matrix $\left\{{x^{\prime }}_{ij}\right\}.$ Subsequently, we relocate the standardized formula to the right by 0.001 units to simplify the subsequent logarithmic calculations.

(2) $$x_{ij}=\frac{{x^{\prime }}_{ij}-\min\left({x^{\prime }}_{j}\right)}{\max\left({x^{\prime }}_{j}\right)-\min\left({x^{\prime }}_{j}\right)}+0.001,\;where\;i=1,2,3,\ldots ,n,\;and\;j=1,2,3,\ldots ,m.$$

The entropy value, represented as *e_j_*, has been acquired using Equation (4).

(3) $$r_{ij}=\frac{x_{ij}}{\sum_{i=1}^{n}x_{ij}},\;i=1,2,3,\ldots ,n,\;and\;j=1,2,3,\ldots ,m.$$

(4) $$e_{j}=-\frac{1}{\ln n}\sum_{i=1}^{n}r_{ij},\;i=1,2,3,\ldots ,n\;and\;j=1,2,3,\ldots ,m.$$

The weight $w_{j}$ is calculated using Equation (5).

(5) $$w_{j}=\frac{1-e_{j}}{\sum_{j=1}^{m}\left(1-e_{j}\right)}$$

### 3.2. TOPSIS Model

TOPSIS, which has been introduced by [37], evaluates the extent to which possible solutions approach optimality. The level of proximity is determined by calculating the Euclidean distance between the best and suboptimal solutions for each goal selection. The ideal option is determined by the most favorable value for each evaluation criterion, whereas the unsatisfactory alternative is characterized by the least advantageous value for each assessment criterion [46,47]. We choose the optimum solution as the preferred alternative due to its large divergence from the worst solution and its proximity to the ideal answer. To normalize the dimensions of the criterion, we begin by normalizing the positive and negative criteria of the choice matrix in Equation (1) separately. The use of the min–max approach is essential for achieving consistency across several facets, as shown by Equation (6). This approach streamlines the process of evaluating the pros and cons while computing the entropy weight.

(6) $$positive:x_{ij}^{+}=\frac{{x^{\prime }}_{ij}-\min\left({x^{\prime }}_{j}\right)}{\max\left({x^{\prime }}_{j}\right)-\min\left({x^{\prime }}_{j}\right)}\;negative:x_{ij}^{-}=\frac{\max\left({x^{\prime }}_{j}\right)-{x^{\prime }}_{ij}}{\max\left({x^{\prime }}_{j}\right)-\min in\left({x^{\prime }}_{j}\right)}\;\min\left({x^{\prime }}_{j}\right)=\{\underset{i}{\min}{x^{\prime }}_{ij}\left|1<i<n,1<j<m\right)\}\;\max\left({x^{\prime }}_{j}\right)=\{\underset{i}{\max}{x^{\prime }}_{ij}\left|1<i<n,1<j<m\right)\}$$

The initial choice matrix in Equation (6) is generated by normalizing the positive and negative criteria, resulting in the dimensionless standardized decision matrix *x_ij_*, as illustrated in Equation (7).

(7) $$X^{\prime }=\{\begin{array}{cccc} x_{11} & x_{12} & \cdots & x_{1m}\\ x_{21} & \cdots & \cdots & x_{2m}\\ \vdots & \cdots & \cdots & \vdots \\ x_{n1} & x_{n2} & \cdots & x_{nm} \end{array}\;\},\;where\;i=1,2,3,\ldots ,n\;and\;j=1,2,3,\ldots ,m.$$

Furthermore, the decision matrix in Equation (8) is derived by multiplying each element $v_{ij}=w_{j}\times x_{ij},$ where $w_{j}=(w_{1},w_{2},w_{3},\ldots ,w_{m}$) is obtained from Equation (5) and meets the condition $\sum_{j=1}^{m}w_{j}=1$. $x_{ij}$ is generated using Equation (7).

(8) $$V=\left[\begin{array}{cccc} v_{11} & v_{12} & \cdots & v_{1m}\\ v_{21} & \cdots & \cdots & v_{2m}\\ \vdots & \cdots & \cdots & \vdots \\ v_{n1} & v_{n2} & \cdots & v_{nm} \end{array}\;\right]=\left[\begin{array}{cccc} w_{1}x_{11} & w_{2}x_{12} & \cdots & w_{m}x_{1m}\\ w_{1}x_{21} & w_{2}x_{22} & \cdots & w_{m}x_{2m}\\ \vdots & \cdots & \cdots & \vdots \\ w_{1}x_{n1} & w_{2}x_{n2} & \cdots & w_{m}x_{nm} \end{array}\;\right]$$

Equation (9) defines the positive ideal solution (*PIS*) as the highest value and the negative ideal solution (*NIS*) as the lowest value for each criterion. Moreover, Equations (10) and (11) are used to calculate the distance between each option and the positive ideal solution (*PIS*) and negative ideal solution (*NIS*), respectively.

(9) $$PIS:P^{+}=\left\{v_{1}^{+},v_{2}^{+},v_{3}^{+},\ldots ,\;v_{m}^{+}\right\}=\{(\underset{i}{\max}v_{ij}|j\in M)\}\;NIS:P^{-}=\left\{v_{1}^{-},v_{2}^{-},v_{3}^{-},\ldots ,\;v_{m}^{-}\right\}=\{(\underset{i}{\min}v_{ij}|j\in M)\}$$

(10) $$d_{i}^{+}=\sqrt{\sum_{j=1}^{m}(v_{ij}-v_{j}^{+}{)}^{2}},\;i=1,2,3,\ldots ,n,\;and\;j=1,2,3,\ldots ,m.$$

(11) $$d_{i}^{-}=\sqrt{\sum_{j=1}^{m}(v_{ij}-v_{j}^{-}{)}^{2}},\;i=1,2,3,\ldots ,n,\;and\;j=1,2,3,\ldots ,m.$$

Ultimately, calculate the coefficient of relative closeness (*RC*).

(12) $$RC_{i}=\frac{d_{i}^{-}}{d_{i}^{-}+d_{i}^{+}},\;i=1,2,3,\ldots ,\;n$$

## 4. Results

To evaluate the factors and affect FTT symptoms, we considered five criteria (Table 1). The selected variables and data have been retrieved from the database of UNICEF.

In conclusion, the decision matrix that was generated was employed to conduct the analysis of the study, which can be categorized into three primary sections. Various periods and their respective stages are outlined in the subsequent sections. Initially, the N1 approach is employed to normalize the data in the decision matrix. The objective of this approach is to standardize the data to facilitate comparison. A normalized matrix was generated for each Ni after Step 1 was finished. Table A1 and Table A2 in Appendix A contain the normalized matrices. Additionally, the subsequent phase of the analysis involved the assignment of weights to each criterion *w_ij_* and the generation of the weighted normalized matrix in accordance with the methodology outlined in Section 3.1 of the current study. In this investigation, we implemented the entropy-weight TOPSIS methodology. The weights assigned to each selected criterion are detailed in Table 2.

The Euclidean distance between the positive ideal solution (PIS) and the negative ideal solution (NIS) was subsequently determined. Equations (10) and (11) determine the normalized Euclidean distance for both positive and negative solutions of each choice. The results are depicted in Table 3.

By using the entropy-weight TOPSIS method, it was highlighted that criterion C5 (mothers with higher education level) and criterion one (unhealthy feeding habits in urban) have the most weight and the biggest effect on FTT symptoms in children between 6 and 12 months.

In recent years, there has been a significant focus on studying the impact of mothers’ education on child health, mostly because of its policy implications. Researchers have contrasting opinions on the impact of maternal education on child health, displaying a moderate degree of disagreement. Mothers, being the main caretakers, possess complete control in promoting nutritious eating habits and tending to the needs of their children. However, the present research recognizes the substantial influence of mothers’ education in decreasing FTT among mothers in Sub-Saharan Africa [26]. Furthermore, mothers who possess a higher level of education and extensive knowledge exhibit greater consciousness for the well-being of their offspring.

It is well recognized that mothers with higher levels of education possess a more profound understanding of nutrition, which may be beneficial for promoting healthy eating habits. Mothers who have attained a higher level of education are more inclined to use healthcare facilities to meet their children’s healthcare requirements, particularly about nutrition, notably by giving a greater quantity of food [25]. Moreover, a mother’s inadequate complementary feeding behavior, caused by nutritional deficiencies, lack of knowledge about optimal feeding practices, insufficient awareness of feeding frequency and portion sizes for the child, and unfamiliarity with a balanced diet and cultural beliefs, are changeable factors that can lead to a decline in a child’s nutritional condition. Furthermore, the World Health Organization (WHO) advises that malnourished pregnant women get nutritional instruction and increase their daily intake of calories and protein to decrease the likelihood of giving birth to babies with low birth weight. Consuming well-balanced energy and protein supplements might potentially mitigate insufficient nutrition in women, hence decreasing adverse pregnancy outcomes.

## 5. Discussion and Conclusions

Primary care doctors often encounter the medical problem referred to as failure to thrive. Prompt intervention and prompt diagnosis are crucial to avoid malnutrition and its related developmental problems. If regular exams do not carefully monitor development markers, a considerable percentage of newborns affected by failure to thrive (FTT) go unnoticed [48,49]. An extensive historical analysis is the most efficient approach to identify the underlying cause of the FTT and provide guidance for further evaluation and treatment. In cases of catch-up development, it is necessary for all children diagnosed with failure to thrive to consume an extra 150 percent of the caloric requirement, which is determined based on their projected weight rather than their actual weight. Only a small number of cases need laboratory examination. Hospitalization is reserved for patients who are at risk of harm or who show significant failure to thrive. We recommend using a comprehensive strategy in cases when there is persistent severe failure to thrive despite intervention.

Regarding the cause of FTT, more research has shown that stress and other psychosocial variables often play a role in this medical disease. Moreover, most studies provide evidence that symptoms of FTT are more prevalent among newborns residing in rural regions. The primary aim of the present research is two-fold: (i) to examine additional variables associated with FTT and (ii) to assess their influence on FTT in Sub-Saharan African nations and their metropolitan regions. This research is distinctive because it not only discovers supplementary components associated with FTT but also provides a numerical value to each aspect, so emphasizing the dynamic and intensive character of the indicators. We accomplished this by using the entropy-weight and TOPSIS methodologies.

We have chosen five parameters to assess the FTT phenomena in nine countries located in the Sub-Saharan region. We obtained the criteria and corresponding statistics from the UNICEF database. The criteria consisted of (i) poor eating habits in urban regions; (ii) maternal education, no Education; (iii) maternal education, primary education; (iv) maternal education, secondary education; and (v) maternal education: higher education. The findings suggest that maternal education, particularly at a higher level, has the most significant impact. In the last two decades, there has been substantial advancement in the enrollment and graduation rates of both women and men in higher education worldwide, especially in Sub-Saharan Africa. Nevertheless, disparities persist in the extent of participation between women in the higher education systems of African nations. Inequalities are particularly evident in several domains, including the unequal distribution of higher degree completions, disparities in the choices of subjects of study between men and women, and the enduring gender gaps in academic and senior executive posts within institutions. Advocating for gender equality in higher education stimulates innovation, generates avenues for women’s financial independence, and brings broader social benefits by challenging traditional gender norms. In addition, encouraging moms to pursue higher education may aid in preventing newborn nutritional imbalances and indications of FTT. In addition, it is crucial to promote knowledge and understanding of healthy eating habits and lifestyles among women, especially those who are overweight or obese, to reduce the chances of them having children who are overweight.

Moreover, the second most important factor, which carries greater weight, is the presence of detrimental behaviors, particularly the total lack of fruit and vegetable intake among toddlers aged 6–12 months. Fruits and vegetables are vital sources of a diverse array of macronutrients and micronutrients. Prior research has shown that the consumption of fruits and vegetables is linked to a reduced probability of acquiring chronic diseases. Young individuals who consume more fruits and vegetables have a higher intake of a wide range of essential nutrients, both in larger quantities (macronutrients) and in smaller quantities (micronutrients). As a result, there is an enhancement in the individual’s nutritional condition. Conversely, a diet that lacks diversity in food choices is linked to poor physical growth, impaired cognitive development, and FTT. Within the region of Sub-Saharan Africa, the intake of fruits and vegetables is initially minimal during the period of 6 to 12 months, but it progressively rises as the children age. Throughout the country, there was a significant lack of fruit and vegetable eating, with this infrequent occurrence consistently noted among persons of all age groups. Although not all youngsters take fruit on a regular basis, they generally consume veggies around once per day on average. Notably, undernourished children with insufficient development patterns consumed a lower number of fruits and vegetables, both in terms of quantity and frequency. We have yet to completely comprehend the exact processes by which fruits and vegetables contribute to the prevention of FTT.

Hence, a potential study proposal may include a comprehensive examination of the exact processes used by fruits and vegetables to mitigate FTT, a phenomenon that remains incompletely elucidated. Moreover, future inquiries should give priority to investigating other maternal factors that might potentially lead to FTT. Variables such as marital status, parity, ethnicity, and mothers’ health awareness and health-related behavior, including pregnancy goals, may be used as indications for family planning. Likewise, we may use the location of delivery as a determinant for the act of seeking healthcare. A strong correlation was seen between these characteristics and child FTT.

## Figures and Tables

**Table 1:** Criteria and their computation units.

Criteria	Description	Measurement
C1	Unhealthy feeding habits in urban	Children 6–12 months of age who did not consume any vegetables or fruits
C2	Maternal Education: No Education	Mothers with no education
C3	Maternal Education: Primary Education	Mothers with primary education
C4	Maternal Education: Secondary Education	Mothers with secondary education
C5	Maternal Education: Higher Education	Mothers with higher education

**Table 2:** Entropy-weight TOPSIS approach determines criteria weight.

Criterion	C1	C2	C3	C4	C5
*e_j_*	−0.9443	−0.9789	−0.9558	−0.9638	−0.9077
*D* = 1 − *e_j_*	0.0557	0.0211	0.0442	0.0362	0.0923
*W_j_*	0.2233	0.0846	0.1771	0.1451	0.3699

**Table 3:** Calculation of the Euclidean distance between PIS and NIS.

S_i_^+^	S_i_^−^	Si^+^ Si^−^	Si^−^/(Si^+^ Si^−^)
0.0299	0.1613	0.1912	0.8436
0.0456	0.1535	0.1991	0.7709
0.0274	0.1695	0.1968	0.8610
0.1004	0.0924	0.1928	0.4793
0.1577	0.0392	0.1969	0.1992
0.0765	0.1105	0.1871	0.5909
0.1794	0.0109	0.1902	0.0571
0.1149	0.0755	0.1904	0.3964
0.1670	0.0263	0.1932	0.1359

## Data Availability

The data presented in this study are available upon request from the corresponding author. The data are not publicly available due to privacy and ethical considerations.

## Appendix A

**Table A1:** Normalized matrix.

C1	C2	C3	C4	C5
0.186	0.144	0.165	0.142	0.196
0.163	0.127	0.139	0.166	0.220
0.175	0.174	0.183	0.157	0.158
0.110	0.113	0.129	0.120	0.066
0.072	0.065	0.036	0.076	0.089
0.123	0.115	0.123	0.127	0.156
0.037	0.067	0.054	0.047	0.022
0.095	0.110	0.099	0.118	0.060
0.038	0.084	0.072	0.047	0.033

**Table A2:** Ranking of overall score using TOPSIS method.

Countries	Ranking by FTT Index	Ranking FTT Index by TOPSIS
Burkina Faso	0.7581	0.8436
Chad	0.8372	0.7709
Ethiopia	0.8905	0.8610
Mali	0.7725	0.4793
Niger	0.5248	0.1992
Sao Tome and Principe	0.6925	0.5909
Senegal	0.3142	0.0571
Sierra Leone	0.6653	0.3964
Zimbabwe	0.2521	0.1359
